# Red blood cell transfusion in obstetrics and its implication for patient blood management: a retrospective analysis in Switzerland from 1998 to 2016

**DOI:** 10.1007/s00404-020-05744-7

**Published:** 2020-08-19

**Authors:** Jarmila A. Zdanowicz, Sophie Schneider, Martin Mueller, Ruedi Tschudi, Daniel Surbek

**Affiliations:** 1 Department of Obstetrics and Gynecology, Bern University Hospital, University of Bern, Theodor-Kocher-Haus, Friedbühlstrasse 19, 3010 Bern, Switzerland; 2 Sevisa AG, Ermatingen, Switzerland

**Keywords:** Patient blood management, Peripartum hemorrhage, Red blood cell, Transfusion

## Abstract

**Purpose:**

Peripartum hemorrhage (PPH) remains one of the main causes of maternal mortality worldwide. Treatment includes administration of packed red blood cells (RBC) in severe cases and patient blood management (PBM) may reduce it significantly. In our study, we wanted to retrospectively assess red blood cell administration in PPH to evaluate the impact of PBM in Switzerland.

**Methods:**

Using data from the Swiss obstetric hospital registry (Arbeitsgemeinschaft Schweizer Frauenkliniken, ASF), we included patients with deliveries from 1998 to 2016. We examined available obstetric data as well as blood loss and RBC administration in the acute and subacute peripartal phase. We categorized data into two time intervals: 1998–2011 and 2012–2016, as new PPH guidelines in Switzerland were established in 2012.

**Results:**

PPH incidence increased between 1998 and 2016 significantly. The number of vaginal instrumental deliveries and cesarean sections increased as well. Administration of three or more RBC units, as defined in the ASF registry, in the acute and subacute phase in Switzerland has decreased after 2012. Conversely, we saw an increase in the administration of one to two RBC units in the acute and subacute phase. Nevertheless, overall RBC administration has been decreasing from 1998 to 2016.

**Conclusion:**

The increase of patients obtaining one or two units of RBC for PPH suggests that there may be a potential for effective implication of PBM in obstetrics. Reduction of RBC transfusion in the context of PPH may not only decrease maternal morbidity, but decrease economic costs as well.

## Introduction

Peripartum hemorrhage (PPH) remains one of the leading causes of maternal mortality worldwide, with an incidence of more than 15% [1, 2]. According to the World Health Organization (WHO), PPH is defined as a blood loss of more than 500 ml within 24 h after delivery [3]. In German-speaking countries, including Switzerland, Germany and Austria, PPH is defined as a blood loss of at least 500 ml after vaginal delivery and of at least 1000 ml after Cesarean section (CS) [4].

Severe PPH usually requires the administration of red blood cell (RBC) transfusions. Yet, the administration of RBC itself has been shown to be associated with complications for the patient. Specific transfusion risks include transmission of infections, hemolytic transfusion reactions, transfusion-associated circulatory overload as well as transfusion-associated acute lung injury [5, 6]. But it can also lead to infections, ischemic events as well as multisystem organ failure [7]. In addition, several studies have shown that there are advantages to a restrictive use of RBC transfusions, including better clinical outcomes for patients, decreased morbidity and mortality, shorter hospitalization length and a lower risk for admission to the intensive care unit [7, 8].

Not surprisingly, other medical professions such as trauma or cardiothoracic surgeons introduced guidelines for restrictive RBC administration, which is part of patient blood management (PBM). PBM is based on three pillars, aiming to (1) identify and treat anemia, (2) reduce blood loss and (3) reduce RBC administration [7, 9]. Several studies have shown that successful PBM leads to a quicker patient recovery and fewer postoperative complications [10–13]. To date, only few countries have implemented strategies for effective patient blood management in obstetrics.

In Western Australia, the PBM program has been implemented since 2008 and has shown to reduce hospital mortality, average hospital length of stay and hospital-acquired infections by one-fourth, respectively [14]. In 2015, PBM was developed specifically for the obstetric clinical practice [15].

The most common obstetric risk factors for PPH include placental pathologies (including abnormally invasive placenta, retrained placenta or placental abruption), previous PPH, high maternal parity and pre-eclampsia, but also anemia and antepartum hemorrhage [4, 16, 17]. A further categorization of PPH can be made into primary or acute bleeding within 24 h of delivery, and secondary or subacute, which is bleeding after 24 h up to 12 weeks after delivery [18].

While some of the risk factors for PPH can be assessed during pregnancy and prior to delivery, up to two-thirds of women with a PPH have no known risk factors [19]. However, iron deficiency anemia can be assessed easily. In Switzerland, new guidelines for treating anemia in pregnancy were implemented in 2012 and recently updated [20]. Severe anemia in pregnancy is associated with fetal and maternal morbidities such as fetal growth restriction and preterm birth [21, 22]. In addition, anemia itself is a known risk factor for PPH.

Multiple obstetric guidelines exist for PPH management, while in Switzerland, Germany, and Austria an universal guideline for PPH treatment was introduced by obstetric societies [4]. One important update of those guidelines occurred in 2012, re-evaluating the application of conservative and surgical treatment of PPH, including RBC administration [23].

The aim of our study was to examine red blood cell transfusions in Switzerland over the last 18 years, using a large retrospectively database. Specifically, we studied the impact of new PPH guidelines on the administration of RBC.

## Materials and methods

This was a retrospective database study using data from the Swiss obstetric hospital registry (Arbeitsgemeinschaft Schweizer Frauenkliniken, ASF) which is managed by Sevisa AG. This registry includes anonymized patient delivery and obstetric data from 40 obstetric hospitals, including primary, secondary and tertiary units, in Switzerland. Most hospitals included in this database are primary and secondary unit obstetric hospitals. Data are collected by physicians in each involved hospital and subsequently submitted and analyzed by Sevisa AG.

Patients who gave birth between January 1, 1998, and December 31, 2016, were included. We looked at RBC administration in the acute and subacute phase, blood loss, hemoglobin levels, potential PPH risk factors, need for postpartum intensive care unit (ICU) hospitalization, delivery mode and overall obstetric data. The amount of blood loss was determined at the discretion of each hospital (usually using visual estimates, weight of pads and surgical cloths, and surgical containers). Regarding RBC administration, we looked at overall RBC administration during hospitalization, as well as detailed data regarding administration of one or two RBC unit versus more three units or more (as used in the ASF data registry) in the acute and subacute phase during hospitalization after delivery.

Data were collected using administrative data. As new guidelines on PPH treatment were established in 2012 (as described above) and expected to be implemented by participating hospitals, we categorized data into two time intervals: 1998–2011 and 2012–2016. In addition, deliveries were categorized according to delivery mode: spontaneous vaginal birth, instrumental vaginal delivery and Cesarean section.

### Statistical analysis

This analysis was based on annual reports from 1998 to 2016 that contained counts of categorical variables and means with standard deviation (SD) of continuous variables, hence on aggregated data. We pooled these data to achieve summary data for the two periods 1998–2011 and 2012–2016, respectively. To investigate whether these periods were different, we calculated pooled counts and % of the categorical variables per period and compared the proportions by calculating odds ratios with 95% confidence intervals and exact *p* values. We also calculated pooled means and SDs for maternal age, gestational age and neonatal weight und used *T* tests to investigate differences. To assess whether there was a trend with respect to the absolute number of deliveries and relative number of different types of deliveries, we calculated Spearman’s rho with corresponding *p* value. All analyses were performed using Stata 14 (Stata Corp., College Station, Texas). A *p* value < 0.05 was considered significant.

## Results

We included a total of 627,921 deliveries from 1998 to 2016.

**Fig. 1:**
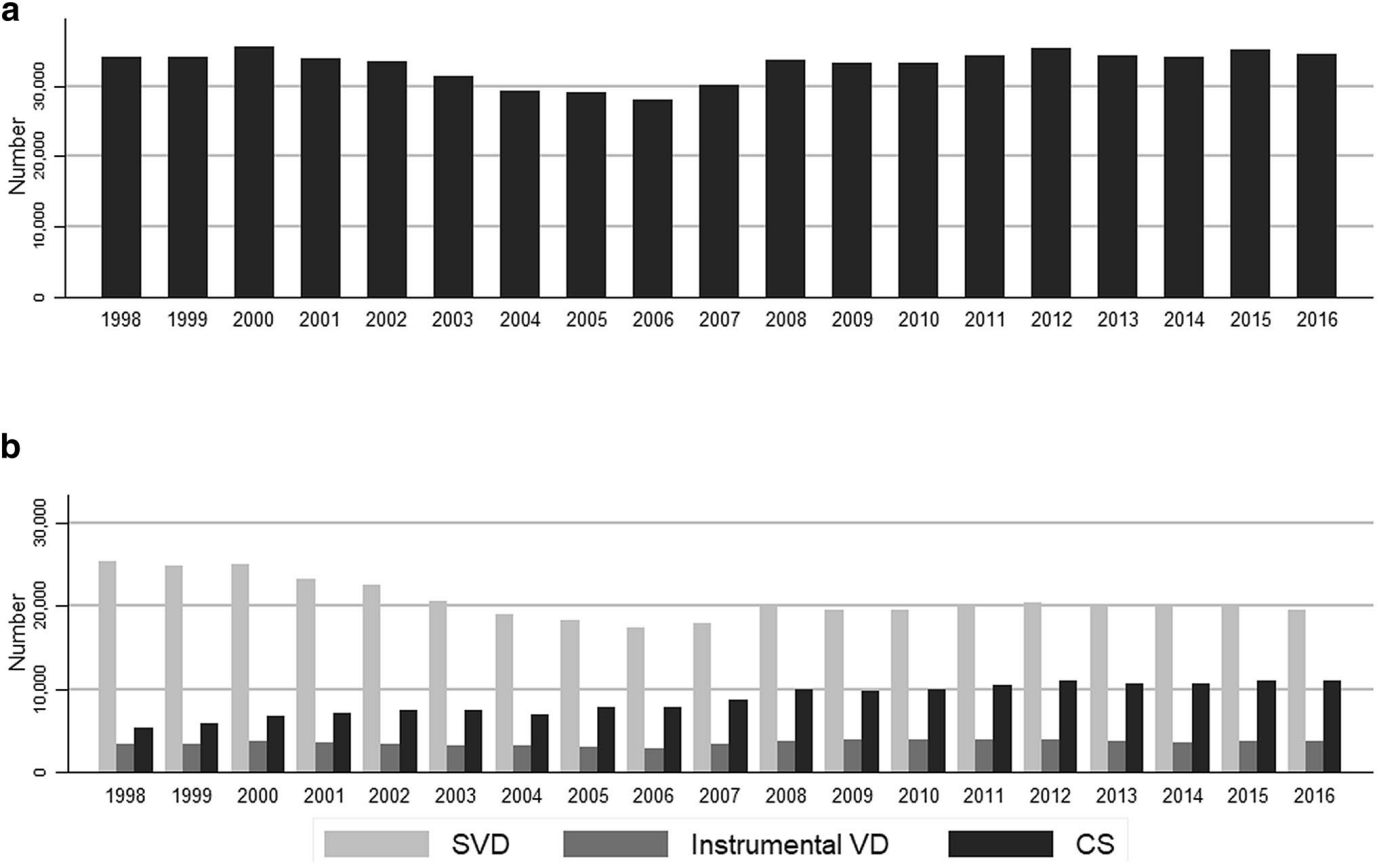
**a** Total number of deliveries from 1998–2016; **b** deliveries from 1998–2016 according to delivery mode (*SVD* spontaneous vaginal delivery, *Instrumental VD* instrumental vaginal delivery, *CS* Cesarean section)

From 1998 to 2016, there was an increase in cesarean section as well as instrumental deliveries and a decrease in spontaneous vaginal delivery (Fig. 1).

From 1998 to 2011, 454,463 deliveries were included, while from 2012 to 2016 173,458 deliveries were included. Peripartal data for both time intervals are summarized in Table 1.

**Table 1:** Comparison of peripartal outcome and risk factors for peripartum hemorrhage from 1998–2011 and 2012–2016 (*SD* standard deviation, *ICU* intensive care unit, *RBC* red blood cell, *Hb* hemoglobin)

Total *N* = 627,921	1998–2011	2012–2016	Odds ratio (95% CI)	*p* value
	*N* = 453,463	*N* = 173,458		
Mean maternal age (years) ± SD	30.0 ± 5.0	31.0 ± 4.9		< 0.001
Mean length of stay (days)	5.4	4.6		
Mean gestational age (weeks) ± SD	39 1/7 ± 18 days	39 1/7 ± 18 days		< 0.001
Mean birth weight (g) ± SD	3349 ± 504	3330 ± 507		< 0.001
Required ICU visit	1194 (0.3%)	676 (0.4%)	1.48 (1.35–1.63)	< 0.001
Peripartum (acute phase)				
Intraoperative blood loss 500–1000 ml	40,842 (9.0%)	22,975 (13.2%)	1.54 (1.52–1.57)	< 0.001
Intraoperative blood loss > 1000 ml	5149 (1.1%)	3716 (2.1%)	1.91 (1.83–1.99)	< 0.001
Peripartum RBC units (acute)				
Intraoperative 1–2 RBC units	913 (0.2%)	444 (0.3%)	1.27 (1.13–1.43)	< 0.001
Intraoperative > 2 RBC units	730 (0.2%)	250 (0.1%)	0.90 (0.77–1.04)	0.134
Postoperative 1–2 RBC units	880 (0.2%)	425 (0.2%)	1.26 (1.12–1.42)	< 0.001
Postoperative > 2 RBC units	558 (0.1%)	180 (0.1%)	0.84 (0.71–1.00)	0.048
Hospital stay after birth (subacute)				
Hb < 100 g/l	26,431 (5.8%)	5274 (3.0%)	0.51 (0.49–0.52)	< 0.001
RBC units (subacute)				
1–2 RBC units	1871 (0.4%)	953 (0.5%)	1.33 (1.23–1.44)	< 0.001
3 RBC units or more	1184 (0.3%)	400 (0.2%)	0.88 (0.79–0.99)	0.033
Possible risk factors				
Retained placental products	8744 (1.9%)	4014 (2.3%)	1.20 (1.16–1.25)	< 0.001
Placenta previa	1275 (0.3%)	654 (0.4%)	1.34 (1.22–1.48)	< 0.001
Uterine rupture	129 (0.0%)	84 (0.0%)	1.70 (1.28–2.26)	< 0.001
Previous Cesarean section	47,646 (10.5%)	24,409 (14.1%)	1.39 (1.37–1.42)	< 0.001
Multiple gestation delivery	6208 (1.4%)	3100 (1.8%)	1.31 (1.25–1.37)	< 0.001
Induction of labor	91,335 (20.1%)	36,864 (21.3%)	1.07 (1.06–1.08)	< 0.001
Prolonged labor	27,181 (6.0%)	10,891 (6.3%)	1.05 (1.03–1.08)	< 0.001
Polyhydramnios	2689 (0.6%)	1698 (1.0%)	1.66 (1.56–1.76)	< 0.001
Amniotic infection syndrome	1026 (0.2%)	521 (0.3%)	1.33 (1.19–1.48)	< 0.001
Primary uterine inertia	135,248 (29.8%)	46,106 (26.6%)	0.85 (0.84–0.86)	< 0.001
Placenta accreta/increta/percreta	1558 (0.3%)	593 (0.3%)	1.00 (0.90–1.10)	0.942
Placental abruption	1315 (0.3%)	464 (0.3%)	0.92 (0.83–1.03)	0.137
Pre-eclampsia	5673 (1.3%)	2310 (1.3%)	1.07 (1.01–1.12)	0.011
Low-lying placenta	1635 (0.4%)	648 (0.4%)	1.04 (0.94–1.14)	0.439

Between the two time intervals 1998–2011 and 2012—2016, there was a significant increase in mean maternal age and required intensive care unit (ICU) visit, and a significant decrease in mean birth weight. In addition, there was an increase in potential risk factors for PPH, including retained placental products, placenta previa, uterine rupture, previous cesarean sections, multiple gestation delivery, induction of labor, prolonged labor, polyhydramnios and amniotic infection syndrome. Interestingly, there was no difference in the incidence of abnormal placentation, including placenta accrete, increta or percreta. There was a significant increase in intraoperative blood loss and RBC administration of one to two units in the acute phase. In the subacute phase, there was a significant increase RBC administration of one to two units as well.

**Fig. 2:**
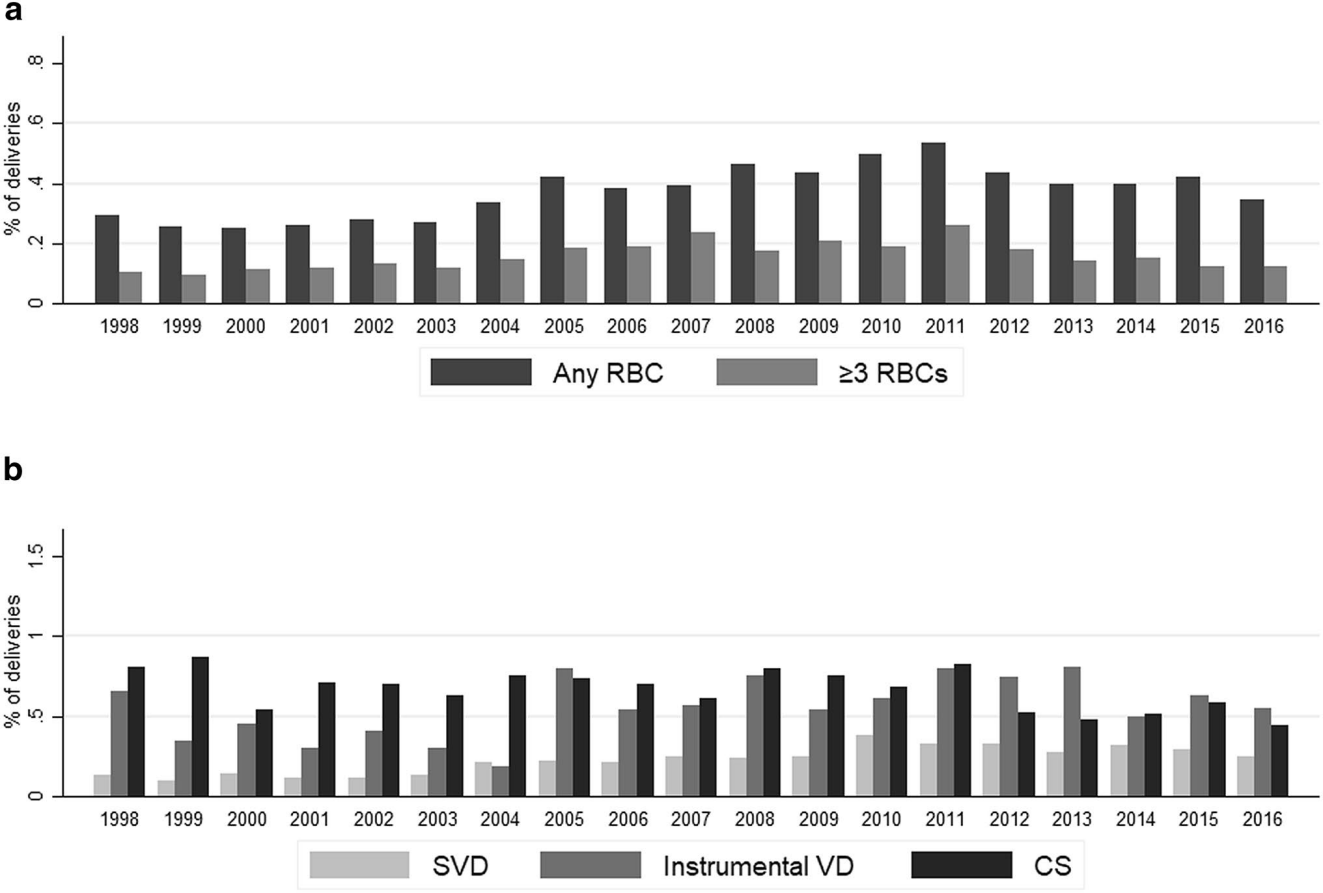
**a** RBC administration in the acute phase from 1998–2016 across all deliveries; **b** overall RBC administration in the acute phase from 1998–2016 across individual delivery modes (*RBC* red blood cell, *SVD* spontaneous vaginal delivery, *Instrumental VD* instrumental vaginal delivery, *CS* Cesarean section)

In the acute phase within 24 h after delivery, there was a decrease in the overall administration of RBC units as well as administration of three or more RBC units across all deliveries after 2012 (Fig. 2a). Instrumental vaginal delivery seems to be associated with an increased need for RBC administration (Fig. 2b).

**Fig. 3:**
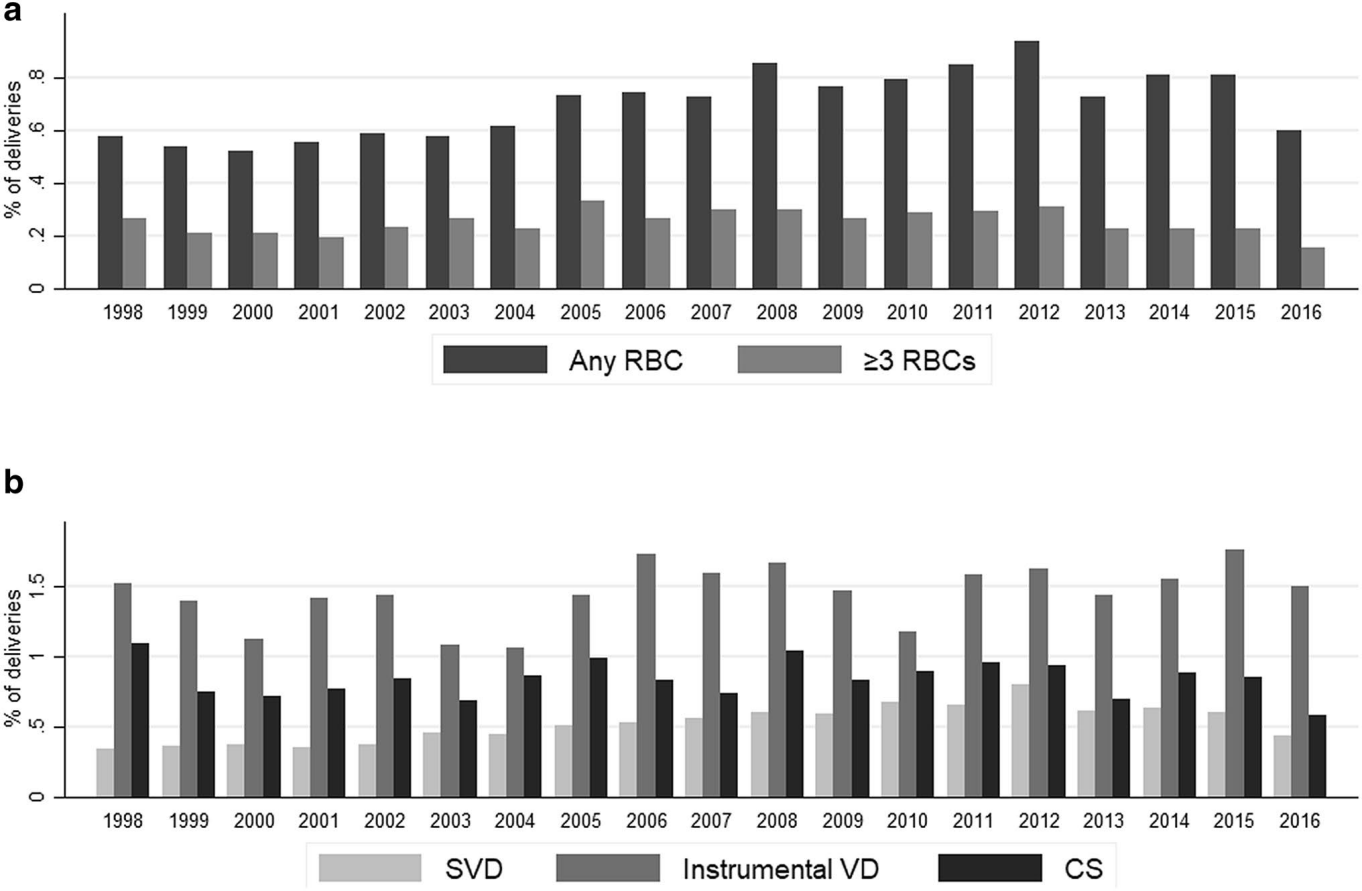
**a** RBC administration in the subacute phase from 1998–2016 across all deliveries; **b** overall RBC administration in the subacute phase from 1998–2016 across individual delivery modes (*RBC* red blood cell, *SVD* spontaneous vaginal delivery, *Instrumental VD* instrumental vaginal delivery, *CS* Cesarean section)

In the subacute phase, there was a decrease of overall RBC administration as well as administration of three RBC units or more (Fig. 3a). In the subacute phase, instrumental vaginal delivery seems to be associated with an increased need for RBC administration as in the acute phase (Fig. 3b).

Overall, the number of three or more RBC units administered has decreased in both acute and subacute phase after 2012, while the number of one to two RBC units administered in the acute or subacute phase has increased after 2012. In addition, as can be seen in Figs. 2a and 3a, instrumental delivery seems to be associated with peripartum hemorrhage and RBC administration in the acute and subacute phase.

## Discussion

As has been previously described, there is a trend towards increasing PPH in the past 20 years in Switzerland, with an increase in vaginal instrumental deliveries and cesarean sections [24]. At the same time, our study shows that despite this increase, excessive RBC administration in the acute and subacute phase in Switzerland has been decreasing after 2012. This is in line with the implementation of Swiss guidelines on PPH and anemia in pregnancy [4, 20, 23]. Nevertheless, there was an increase in the administration of one to two RBC units. Our study has also shown that there has been an increase in risk factors over the years. However, as the majority of women have no known risk factors for PPH, a guideline for patient blood management should be in place which would potentially reduce even low RBC administration. For example, anemia is a major risk factor for PPH [25]. Current Swiss guidelines recommend regular screening of hemoglobin levels at least once per trimester as well as to screen for iron levels in the first trimester [20]. This is one step towards an effective patient blood management.

Yet, recently there has been more focus on acute treatment of PPH with increased early use of tranexamic acid. Several studies have already shown and currently further studies are ongoing about the effective use of tranexamic acid as a first-line therapy or even prophylactic use for PPH [17, 26–29]. Other studies have indicated that the administration of carbetocin as well as oxytocin can effectively prevent PPH after vaginal delivery [30, 31]. In addition, cell saver, which uses allogenic blood, might also benefit women with an increased risk for PPH [32].

The strength of our study is its large sample size, the large time interval of 18 years and the inclusion of 40 hospitals. One weakness of our study is its retrospective character; however, due to the large sample size possibly misrecorded data should be largely counterbalanced. In addition, the two time intervals we chose were not equal in length, the interval from 2012 to 2016 only encompassing five years. Furthermore, as data for the registry is usually collected before placental histology becomes available, this might have been insufficiently recorded on data sheets. In addition, we refrained from calculating a multivariate analysis to investigate possible interactions of risk factors based on our aggregated data as results would not be interpretable because of associations between variables due to ecological fallacy.

Overall, our study shows that there is still a need for providing and optimizing PBM in obstetrics. Common guidelines need to be established to effectively prevent PPH in obstetrics. This might include a standardized questionnaire for PPH risk factors in a prenatal screening, autologous transfusion and, as already mentioned, prophylactic administration of tranexamic acid. PBM is profitable from an economic point of view as well. It is associated with lower hospitalization costs, lower transfusion costs as well as lower in-patient costs.

Currently, PBM in obstetrics and its impact on maternal and perinatal morbidity and mortality is understudied. Furthermore, awareness of PBM in obstetrical units is low, suggesting a great potential for improving the grade of implementation of PBM with a beneficial medical and economic impact.
